# How is the third jaw joint in whales different? Diverse modes of articulation between the jaws of whales

**DOI:** 10.1111/joa.70008

**Published:** 2025-06-18

**Authors:** Rebecca J. Strauch, Nicholas D. Pyenson, Carlos Mauricio Peredo

**Affiliations:** ^1^ Department of Geology and Environmental Earth Science Miami University Oxford Ohio USA; ^2^ Department of Paleobiology, National Museum of Natural History Smithsonian Institution Washington District of Columbia USA; ^3^ Department of Institutional Analytics and Research Western Governors University Salt Lake City Utah USA

**Keywords:** aquatic feeding, cetaceans, evolution, joint morphology, mandible, mandibular symphysis, osteology, symphyseal fusion

## Abstract

Cetaceans are a lineage of marine mammals that evolved diverse modes of aquatic feeding facilitated by modifications to the ancestral mammalian feeding apparatus, including the mandibular symphysis. In mammals, the mandibular symphysis is the third joint of the lower jaw. Articulation of the joint varies across mammalian clades, ranging from fibrocartilaginous connection to complete ossification. Whales span this range, with one lineage (baleen whales) evolving an unfused, highly mobile symphysis. This study conducts a comprehensive morphological investigation of the mandibular symphysis in whales. Here, we describe diverse joint morphologies based on observations of 152 cetacean mandibles representing 74 extant and fossil taxa. We also examine the internal architecture of the joint using computed tomography (CT) cross‐sectional data. Based on gross anatomical observations of the osteology of the joint, we define four broad categories of symphyses: unfused, partially fused, fully fused, and decoupled. In odontocetes, articulation ranges from unfused mandibles to full fusion of the symphysis. The decoupled, highly mobile symphysis in crown mysticetes represents a novel condition unobserved in other mammalian clades. Partial fusion of the symphysis is the most common mode of articulation among the observed extant taxa, closely followed by unfused symphyses. In extant and extinct longirostrine taxa, full fusion coincides with an elongated symphysis. However, extant sperm whales (*Physeter macrocephalus*) notably exhibit an elongated, unfused symphysis that likely does not play a significant role in feeding. Observations of eminences on the posterior border of the symphysis in sperm whales and other suction feeders suggest that aspects of hyolingual musculature and function may be reflected in the morphology of symphysis. We suggest that further investigation of the symphyseal joint in marine mammals and other aquatic tetrapods will advance efforts to identify phylogenetic and ecological influences on the form and function of the feeding apparatus in an aquatic environment.

## Graphical abstract

This study conducts the first comprehensive morphological investigation of the mandibular symphysis in whales. Using gross anatomical observation and CT cross‐sectional data, we describe diverse joint morphologies across 74 extant and fossil cetacean taxa. Toothed whales exhibit unfused, partially fused, or fully fused symphyses. Baleen whales evolved a decoupled, highly mobile symphysis that represents a novel condition unobserved in other mammalian clades.

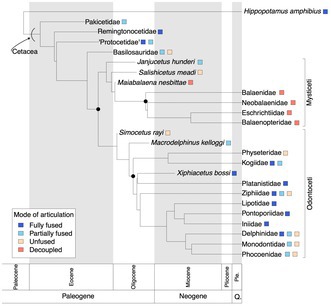

## 1 INTRODUCTION

**FIGURE 1:**
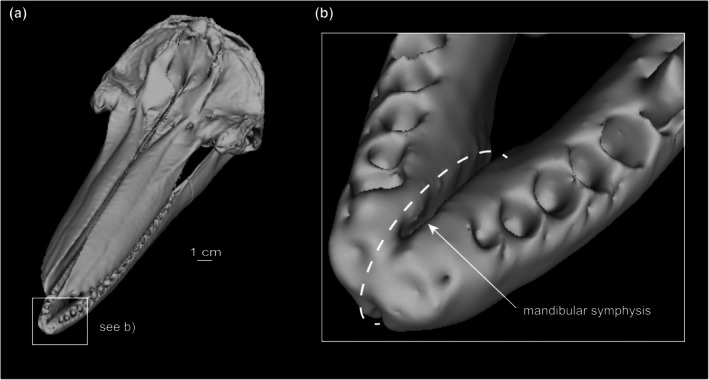
(a) Skull and mandible of *Tursiops truncatus* (SDSNH 21212) in oblique view. (b) Close‐up of mandibular symphysis.

In mammals, the mandibular symphysis is where left and right mandibles articulate anteriorly at the midline (Scapino, 1965, 1981) (Figure 1). Unlike the jaws of other vertebrates, which are made up of multiple bony elements, the mammalian mandible is composed of a single bone: The dentary (Barghusen & Hopson, 1970; Crompton, 1963; Scapino, 1965). As a result, the mandibular symphysis is the only joint between elements of the mandibular apparatus and has been called the third joint of the mammalian masticatory system (Ravosa & Vinyard, 2020; Scapino, 1965). This joint displays a broad range in structure and mobility, resulting in diverse modes of articulation. Some mammals exhibit unfused, kinetic mandibles joined by fibrocartilage and ligaments (Ferreira‐Cardoso et al., 2020). Others have partially or fully fused mandibles with varying degrees of bony interdigitation (Ravosa & Vinyard, 2020). Here, we document and describe the diverse modes of articulation observed in whales (both stem and crown Cetacea).

Cetaceans are a diverse group of marine mammals well‐known for exhibiting extreme modifications to the mammalian body plan inherited from their terrestrial ancestors. Modifications to the feeding apparatus, such as tooth count, rostral length, and mandibular morphology, facilitate diverse modes of aquatic feeding. The mandibles of both baleen whales (mysticetes) and toothed whales (odontocetes) are morphologically specialized for their respective feeding ecologies. In extant mysticetes, elongated, edentulous mandibles coincide with bulk filter feeding (Goldbogen et al., 2017; Pyenson et al., 2013). In odontocetes, the enlarged mandibular fossa, hypertrophied fat bodies, and thin lateral wall of the acoustic window receive and amplify ultrasonic frequencies during echolocation (Cranford et al., 2008; Norris, 1968; Nummela et al., 2007). Morphological specializations at the mandibular symphysis result in novel and diverse modes of articulation in whales relative to other mammalian clades (McCurry & Pyenson, 2019).

Scapino (1981) defined four classes of mandibular symphyses based on the topography of the articulating surfaces and the arrangement of connective tissue filling the symphyseal space. Although this classification system was initially developed for carnivorans (Scapino, 1981), it has been applied to other mammalian (Lee et al., 2019; Ravosa & Vinyard, 2020; Scott et al., 2012; Stover et al., 2017) and nonmammalian clades (Holliday & Nesbitt, 2013; Lessner et al., 2019). In terrestrial mammals, symphyseal morphology has been tied to the biomechanics of mastication, tooth occlusion, and bite force (Bhullar et al., 2019; Ravosa & Vinyard, 2020; Tseng et al., 2016). However, the form and function of the symphysis are less well‐studied in aquatic mammals that do not masticate or process prey using their teeth (Jones et al., 2013). Cetaceans provide an opportunity to study the morphology of the symphysis as it relates to feeding in a novel environment (i.e. aquatic) with clear departures from the ancestral feeding mode (i.e. mastication).

Whales exhibit a wide range of symphyseal morphologies, including extreme departures from the ancestral mammalian condition. In mysticetes, a flexible symphysis permits mandibular rotation and expansion of the oral cavity (Goldbogen et al., 2017; Lambertsen et al., 1995). The onset of this condition in the Oligocene is therefore recognized as an important step in the evolution of filter‐feeding (Fitzgerald, 2012). In odontocetes, the extremely elongated, fully fused symphyses of extant river dolphins and extinct longirostrine taxa exemplify convergence on similar feeding ecologies, such as predation on fast‐moving prey items in a fluid environment (McCurry & Pyenson, 2019). These two extremes—unfused mandibles in baleen whales and fully fused, elongated symphyses in river dolphins—clearly represent adaptations for aquatic feeding. However, the morphology of the symphysis has received less attention in other taxonomic groups, such as oceanic dolphins and beaked whales. As a result, there is a significant research gap in our understanding of how the form and function of the symphysis varies across the full range of feeding behaviors present in whales.

In cetaceans, symphyseal morphology has long been recognized as a diagnostic character trait, with more recent work incorporating symphyseal fusion as a morphological character in phylogenetic analyses (Fitzgerald, 2006; Fordyce, 1994; Geisler & Sanders, 2003). However, phylogenetic scorings used for diagnosing clades do not necessarily capture articular form and function. Few studies have documented the composition and arrangement of connective tissue at the cetacean mandibular symphysis. The relatively recent discovery of a sensory organ in the mandibular symphysis of rorquals demonstrates the value of and need for more anatomical and histological work (Pyenson et al., 2012).

Here, we conduct the first comprehensive morphological investigation of the symphyseal joint in whales. The question motivating this study is twofold: How does the morphology of the cetacean mandibular symphysis vary across extant and fossil taxa? How is this range of variation similar and/or different to the range observed in terrestrial mammals? This study addresses the aforementioned research gaps through its broad taxonomic scope and development of a new framework for interpreting symphyseal form and function in whales. We describe diverse morphologies of the cetacean mandibular symphysis and link modes of articulation to the associated osteology of the joint. We also briefly discuss functional implications of the joint and outstanding questions.

## 2 MATERIALS AND METHODS

### 2.1 Institutional abbreviations

CMNH, Cleveland Museum of Natural History, Cleveland, Ohio, USA; DFO, collections in the Department of Zoology, University of British Columbia, Vancouver, British Columbia, Canada; MVZ, Museum of Vertebrate Zoology, University of California, Berkeley, California, USA; SDSNH, San Diego Natural History Museum, San Diego, California, USA; USNM, Departments of Paleobiology and Vertebrate Zoology (Division of Mammals), National Museum of Natural History, Smithsonian Institution, Washington, District of Columbia, USA; UWBM, Burke Museum of Natural History and Culture, University of Washington, Seattle, Washington State, USA.

### 2.2 Taxonomic sampling

The diverse symphyseal morphologies described here are based on observations of 131 extant and 21 fossil cetacean mandibles reposited in collections at the Cleveland Museum of Natural History (CMNH), the Burke Museum of Natural History and Culture (UWBM), and the Smithsonian Institution's National Museum of Natural History (USNM). This dataset represents 55 extant and 19 fossil taxa spanning all major cetacean clades (Tables S1 and S2). Observations of the internal architecture of the symphysis are based on computed tomography (CT) cross‐sectional slices of a common bottlenose dolphin (*Tursiops truncatus*), Ganges river dolphin (*Platanista gangetica*), and humpback whale (*Megaptera novaeangliae*). The figured slices (a, b, c, and d) are not homologous points. CT scans were sourced from the literature and third‐party databases; see references therein for scanning procedures, digital render methodologies, and processing details (Table 1). Scans of SDSNH 21212 were acquired through Digimorph courtesy of Timothy Rowe. Scans of DFO 2408 were provided by Jeremy A. Goldbogen.

**TABLE 1:** List of specimens scanned, scanning facility, number of CT slices, and source of the scanning information.

Taxon	Specimen	Scanning facility	Number of slices	Source
*Tursiops truncatus*	SDSNH 21212	High‐resolution X‐ray computed tomography facility at the University of Texas at Austin	628	Racicot & Colbert (2002)
*Platanista gangetica*	USNM VZ 23456	Smithsonian Institution Bio‐Imaging (SIBIR) Center in the Department of Anthropology at USNM	2718	Boersma et al. (2017)
*Megaptera novaeangliae*	DFO 2408	Vancouver General Hospital	2010	Field et al. (2010)

We sampled taxa that would increase both taxonomic and temporal coverage. To examine morphological variation across different feeding strategies, we sampled taxa from all 14 extant families. We included fossils from the late Eocene through the Miocene to capture evolutionary transitions leading up to and following the rise of crown groups. Because the selection of scanned specimens was limited by size constraints and the availability of pre‐existing CT‐data, we selected specimens that clearly exemplify the modes of articulation described in this study.

### 2.3 Definitions

Symphyseal surface refers to the anteriormost portion of the medial surface of the mandible that articulates at the symphysis (Mead & Fordyce, 2009). Scapino (1965) refers to the articulating surfaces as symphyseal plates (Scapino, 1965, 1981). Here, we use symphyseal surface and symphyseal plate interchangeably. We follow Scapino (1965) in referring to the space between the articulating surfaces as the symphyseal space (Scapino, 1965, 1981). Sutural obliteration refers to extreme sutural closure that occurs during ontogeny and results in the disappearance of a visible line for the suture (Scapino, 1981).

### 2.4 Classification of symphyses

Based on observations of the taxa examined here, we developed a set of descriptive, structural, and functional criteria for distinguishing different modes of articulation present in the cetacean mandibular symphysis. This framework follows Scapino (1981) in categorizing symphyses based on the topography of the symphyseal surface and development of bony rugosities projecting into the symphyseal space. The structure and composition of connective tissue within the joint space are used to infer joint mobility. Complex suturing or complete ossification constrains joint mobility (synarthrosis). Fibrocartilaginous connection permits some mobility (amphiarthrosis), with synovial joints permitting a wider range of movement (diarthrosis) (Hermanson et al., 2018; Ravosa & Vinyard, 2020).

## 3 RESULTS

### 3.1 Unfused symphyses

**FIGURE 2:**
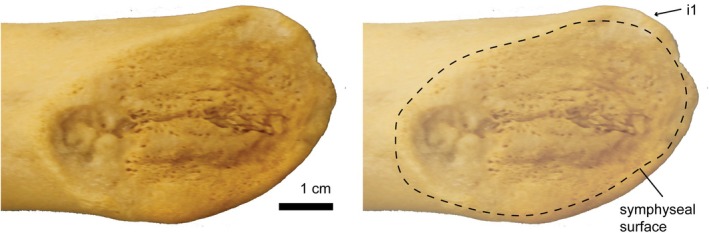
Symphyseal surface of the left mandible of *Phocoenoides dalli* (UWBM 60331). Minimal topography of the surface indicates that mandibles were unfused in life and instead joined by fibrocartilaginous connection. A distinct sulcus that runs along the surface is visible. i1, alveolus of the first incisor.

Unfused symphyses (Class I, see Scapino, 1981) are characterized by a nonsynostotic, fibrocartilaginous suture that may permit some mobility of the joint. This mode of articulation is inferred for 47 specimens representing 21 extant cetacean taxa (Table S3), in which the symphyseal surface ranges from smooth to a modest topography with few low‐lying rugosities (Figure 2). A distinct sulcus or foramen perforates the medial surface in 15 of the observed taxa: *Berardius minimus, Hyperoodon ampullatus, Lissodelphis borealis, Mesoplodon* spp., *Phocoena* spp., *Phocoenoides dalli, Lagenorhynchus obliquidens, Stenella* spp., *Tursiops truncatus*, and *Ziphius cavirostris* (Figure 2; Table S3).

The posterior region of the symphyseal surface is defined by one or two distinct tubercles for attachment of the genioglossus and geniohyoid muscles (Scapino, 1965). We observed a well‐defined posterior border in phocoenids, *Delphinapterus leucas*, ziphiids, and *Physeter macrocephalus*. In the observed specimens of *Phocoena phocoena* and *Phocoenoides dalli*, the raised posterior border is rounded and contributes to the posterior margin of a shallow, circular indentation (Figure 2). In *Phocoena spinipinnis* and *Phocoena sinus*, a notch divides the posterior border into two tubercles, contributing to a heart‐ (*P. spinipinnis*) or arrow‐shaped (*P. sinus*) symphyseal plate. In *Delphinapterus*, a shallow, smooth area separates the tubercle defining the dorsal corner of the posterior border from a small, hook‐shaped eminence on the ventral margin.

Beaked whales, such as *Mesoplodon* and *Ziphius*, exhibit a posterior border delimited by a prominent tubercle. Ventral to the tubercle, the surface of the mandible is excavated by a well‐developed groove that continues anteriorly along the symphyseal surface. In *Ziphius cavirostris*, we observed this groove running beneath the posterior tubercle and above the equally raised ventral margin of the symphyseal surface, dividing the posterior border into two regions.

Of the observed *Ziphius* specimens, three were known juveniles. These specimens exhibit a conspicuously smooth symphyseal surface lacking some of the distinct features observed in the mandibles of ontogenetically older individuals. In USNM VZ 504732 and 504756, the sulcus is less distinct or absent, and the posterior boundary is less well‐developed.

*Physeter* notably exhibits a conspicuously elongated symphysis that is unfused. In the observed female specimens (UWBM 48938, UWBM 48957, UWBM 48961, USNM VZ 395398), the symphyseal surface is relatively smooth with few distinguishable topographic features. A well‐developed groove divides the posterior border of the symphyseal surface into two regions. This groove extends forward as a furrow running just beneath the dorsal margin for at least half the length of the symphyseal plate. In specimens belonging to larger sized adult males (USNM VZ 550876 and 12548), the topography of the symphyseal surface is more complex, with longitudinal ridges spanning the length of the symphyseal plate. The posterior border of the symphysis is defined by well‐developed, rugged eminences tracing the margin of an irregular fossa.

Unfused symphyses are inferred for six of the observed fossil specimens (Table S3). In the right mandible of *Zygorhiza kochii* (USNM PAL 16638), the anterior portion of the symphyseal surface is completely smooth. Long striated ridges span the posterior two‐thirds of the symphyseal surface. An opening likely representing a foramen perforates the surface a quarter of the way from the posterior boundary of the symphyseal plate. The left mandible preserves a similar topography of the symphyseal surface, as well as a foramen. The preserved length of the symphyseal surface (295 and 240 mm in right and left mandibles, respectively) indicates a relatively elongated symphysis spanning more than a third of the mandible's total length (640 and 570 mm preserved in right and left mandibles, respectively).

In *Simocetus rayi*, the smooth symphyseal surface exhibits a slight indentation that may represent a sulcus. Compared with *Zygorhiza*, the symphysis is short, with the length of the symphyseal surface (42 mm) contributing to less than an eighth of the mandible's total length (334 mm preserved). The anterior tip of the mandible is transversally thin, then thickens substantially posterior to the alveolus of i2.

In *Orycterocetus* sp. (USNM PAL 336585), the anterior portion of the symphyseal surface is fenestrated, likely due to taphonomic alteration. Through the fenestrated symphyseal surface, tooth roots are visible extending to the ventral margin of the symphysis. A groove that may represent a longitudinal sulcus divides the smooth, posterior half of the symphyseal surface into two parts. A hole potentially representing a foramen perforates the surface at its posterior boundary. The posterior boundary of the symphyseal surface is vaguely indicated by a change in the slope of the medial surface as the mandible deviates from the sagittal plane. In Physeteridae indet. (USNM PAL 489195), low‐lying ridges run along the symphyseal surface.

### 3.2 Partially fused symphyses

**FIGURE 3:**
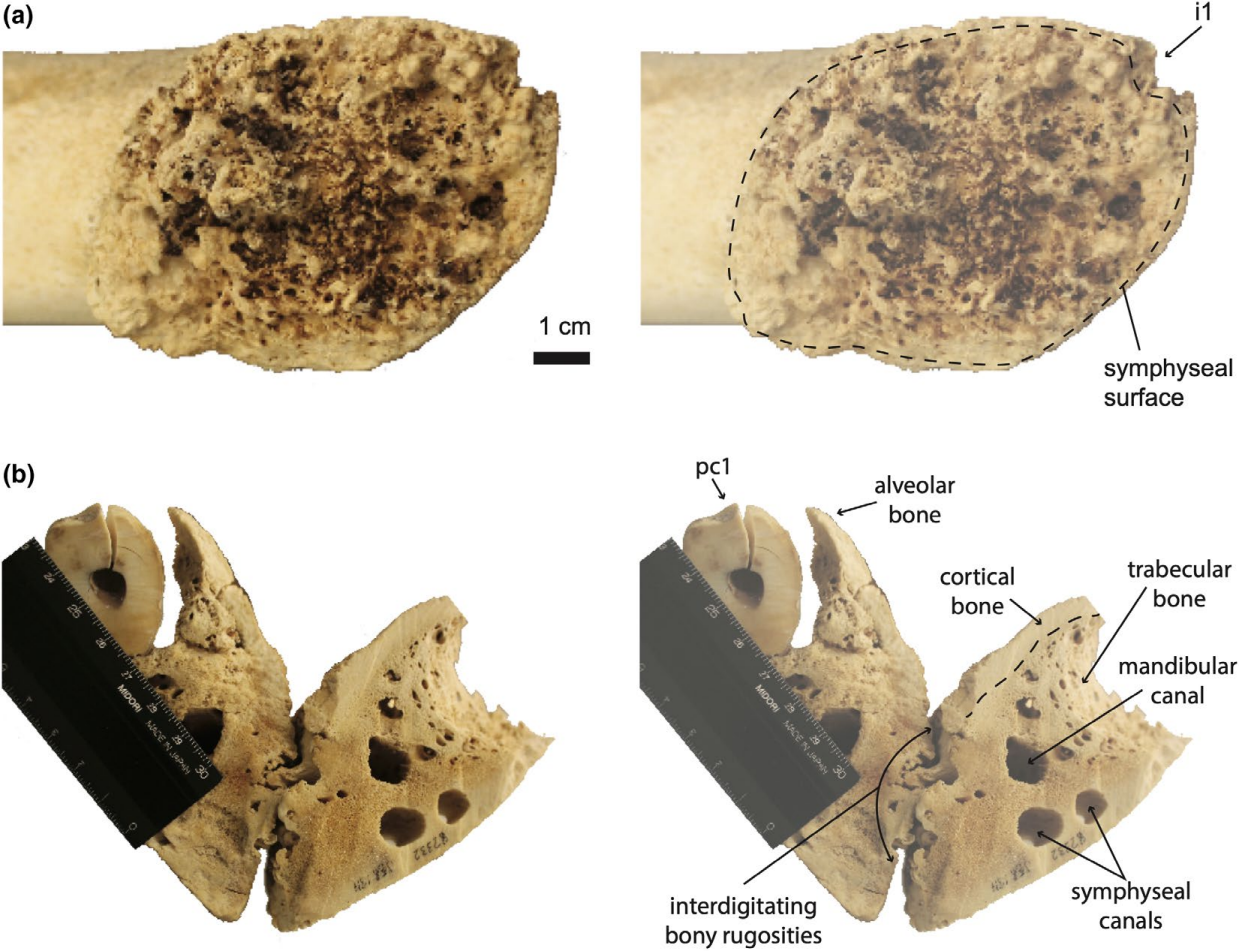
(a) Symphyseal surface of right mandible of *Globicephala macrorhynchus* (UWBM 35429), reflected. Highly rugose topography of the surface indicates that mandibles were partially fused in life and joined by a complex, bony suture. i1, alveolus of the first incisor. (b) Coronal cross‐section (posterior view) of the mandibular symphysis in *Orcinus orca* (UWBM 82332), at the level of the first postcanine. Mandibles are joined by interlocking bony rugosities. Vascularization of the symphyseal region is evinced by the mandibular canal and mental branches of the inferior alveolar canal (symphyseal canals). pc1, first postcanine tooth.

Partially fused symphyses are defined by a synostotic, bony suture with interlocking bony rugosities and calcified ligaments (Figure 3). The functional implication of this morphology is likely an effectively immobile joint. Partial fusion is observed in 59 mandibles representing 29 extant cetacean taxa, for which the symphyseal surface ranges from moderately rugose to significant bony interdigitation (Table S4). In specimens with moderately rugose surfaces (Class II, see Scapino, 1981), distinguishable features of the surface may be observed. In *Berardius bairdii* and *Mesoplodon* spp., we observed sulci running anteroposteriorly along the medial surface. In specimens with highly rugose surfaces (Class III, see Scapino, 1981), rugosities are taller and more numerous (Figure 3a). When mandibles are in firm articulation, these rugosities project into the recesses of the opposite plate and form a complex, bony suture (Figure 3b). Although some sutural obliteration may occur in specimens approaching full fusion, the suture is still visible both dorsally and ventrally for much of the joint's length.

At the anteriormost portion of the symphysis, fusion is typically more extensive dorsally. In several specimens of *Delphinus*, mandibles are fully fused at the anterior tip. In one of the observed specimens of *Feresa attenuata* (USNM VZ 594387), mandibles are nearly fully fused, but the suture is still visible anteriorly, and mandibles begin to pull apart halfway along the symphyseal plate. We observed some sutural obliteration in beaked whales and *Kogia breviceps*, but the sutural obliteration was localized and not extensive.

As in unfused symphyses, the posterior border of the symphyseal surface is defined by eminences for muscle attachment. In delphinids, such as *Globicephala macrorhynchus, Orcinus orca*, and *Peponocephala electra*, the posterior boundary of the symphysis is delimited by a prominent tubercle located near or on the ventral margin of the symphyseal surface. The site of muscle attachment is therefore oriented more ventrally, compared with beaked whales. In *Grampus griseus*, the posterior border is indented by a notch, and the posterior border is defined by two distinct tubercles (as in *Phocoena sinus*).

**FIGURE 4:**
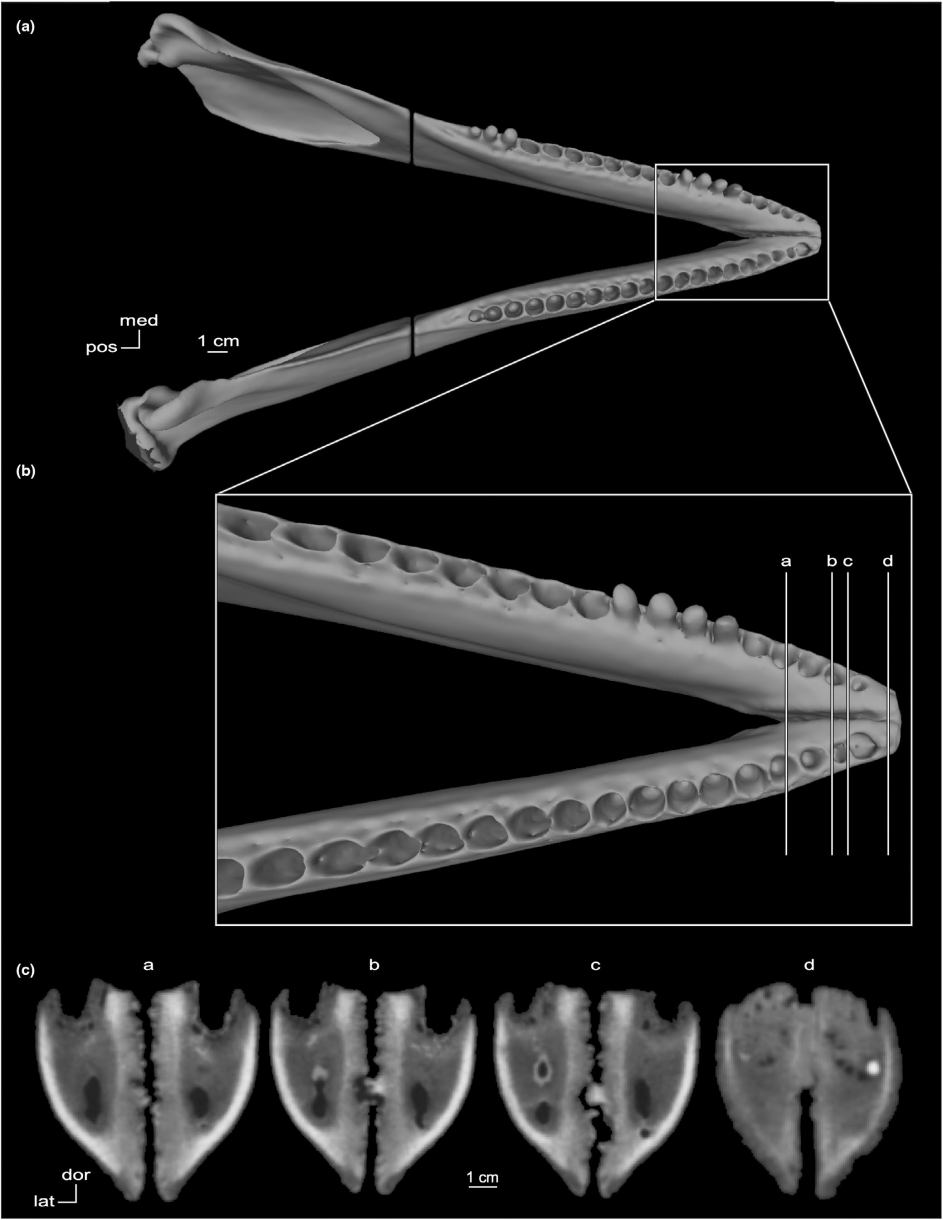
Mandibles of *Tursiops truncatus* (SDSNH 21212). (a) Dorsal view. (b) Close‐up of the symphysis in dorsal view showing the level of CT cross‐sectional slices (a–d). (c) CT coronal cross‐sectional slices (a–d).

CT coronal cross‐sections of *Tursiops truncatus* show bony rugosities projecting into the symphyseal space (Figure 4c). In the posteriormost section (a), the symphyseal space is open (Figure 4c). In intermediate sections (b) and (c), clear interdigitation occurs (Figure 4c). In the anteriormost section (d), a dense region in the dorsal third of the symphysis may indicate bony fusion (Figure 4c). The mandibular canal passes parallel to the symphysis in sections (a‐c), with vascularization of the mandibles continuing into the anteriormost section (d) (Figure 4). In *Orcinus orca*, the symphyseal space is significantly reduced by interdigitating bone (Figure 3b). Fusion is concentrated in the dorsal two‐thirds of the symphysis, with the mandibular canal perhaps constraining fusion in the ventral third. Rugosities develop between the mandibular bodies and not between alveolar bone. The mandibular canal passes parallel to the symphysis. Laterally and ventrally adjacent to the mandibular canal, mental branches of the inferior alveolar canal also pass parallel to the symphysis.

Partially fused symphyses are inferred in five of the observed fossil specimens (Table S4). In *Zygorhiza kochii* (USNM PAL 13774 and USNM PAL V 11962), the anterior half of the symphyseal surface is less rugose than the posterior half. In USNM PAL 13774, a groove likely representing a sulcus runs along the symphyseal surface. In medial view, the posterior boundary is defined by a prominent tubercle at the level of the alveolus of the second premolar. A smaller eminence lies just ventral to this tubercle and also contributes to the posterior border of the symphyseal surface. In USNM PAL V 11962, long striated ridges span the preserved length of the symphysis. In *Janjucetus hunderi* (USNM PAL 534009, cast), the symphyseal surface is short and moderately rugose. The pronounced ventral portion of the posterior border contributes to the posterior boundary of a distinct indentation representing a fossa.

### 3.3 Fully fused symphyses

**FIGURE 5:**
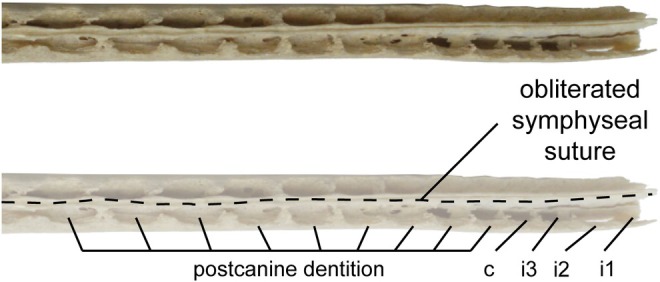
Close‐up of the symphysis of *Platanista gangetica* (USNM VZ 23456) in dorsal view. i1–3, alveoli of incisors; c, alveolus of canine.

Fully fused symphyses (Class IV, see Scapino, 1981) are ankylosed and exhibit extreme synostosis. The functional implication of this morphology is a completely immobile joint. Full fusion is observed in nine specimens representing eight extant cetacean taxa, for which the suture has been obliterated for at least a third of the length of the symphysis (Table S5). A faint line for the suture may still be visible in some specimens, but mandibles cannot be disarticulated without breakage (Figure 5).

*Inia geoffrensis, Lipotes vexillifer, Platanista gangetica*, and *Pontoporia blainvillei* are notable for having extremely elongated symphyses. In *Platanista* and *Pontoporia*, the symphysis is narrow. In *Inia*, the symphyseal region of the mandible is transversally thicker and more robust.

**FIGURE 6:**
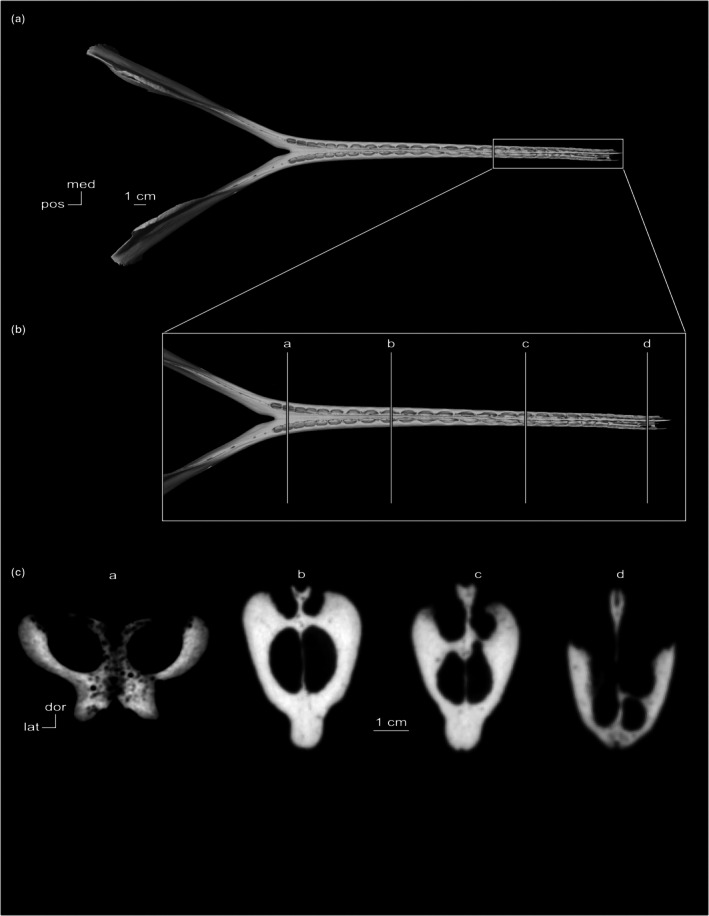
Mandibles of *Platanista gangetica* (USNM VZ 23456). (a) Dorsal view. (b) Close‐up of the symphysis in dorsal view showing the level of CT cross‐sectional slices (a–d). (c) CT coronal cross‐sectional slices (a–d).

In cross‐section, the symphyseal space is completely obliterated. CT coronal cross‐sections of *Platanista* show a thin bony septum at the symphysis (Figure 6c). This septum can be divided into two regions: an interalveolar septum forming a wall between the alveolar canals, and an intermandibular septum forming a wall between the mandibular canals. In the posteriormost section (a), the interalveolar septum disappears as mandibles pull apart from the midline, and the intermandibular septum is retained by less extensive fusion in the joint space (Figure 6c).

We observed full fusion in two beaked whales: *Tasmacetus shepherdi* (USNM VZ 484878, adult female) and *Ziphius cavirostris* (USNM VZ A 21248, adult male). In USNM VZ A 21248, the extent of fusion markedly departs from the unfused symphyses observed in calves and adult females (see Section 3.1). Symphyses approaching full fusion were observed in the other adult male specimens of *Ziphius* (USNM VZ 550064, 550122, and 504856) (see Section 3.2), with sutural obliteration occurring most extensively in USNM VZ A 21248.

We observed fully fused symphyses in seven specimens of the observed fossil taxa (Table S5). In *Araeodelphis, Brevirostrodelphis, Isthminia, Pomatodelphis*, and *Xiphiacetus*, full fusion coincides with an extremely elongated symphysis that contributes to at least half the mandible's total length. In specimens missing anterior portions of the symphysis, such as *Hadrodelphis calvertense* (USNM PAL 23408) and *Brevirostrodelphis dividum* (USNM PAL 21304), a bony septum at the symphysis is visible, as observed in *Platanista* (Figure 6c). In *Araeodelphis, Brevirostrodelphis, Pomatodelphis*, and *Xiphiacetus*, the symphysis is dorsoventrally compressed in cross‐section. In *Isthminia panamensis*, dorsoventral and transverse thicknesses of the symphysis are proportional, similar to the condition observed in *Inia*.

### 3.4 Decoupled symphyses

**FIGURE 7:**
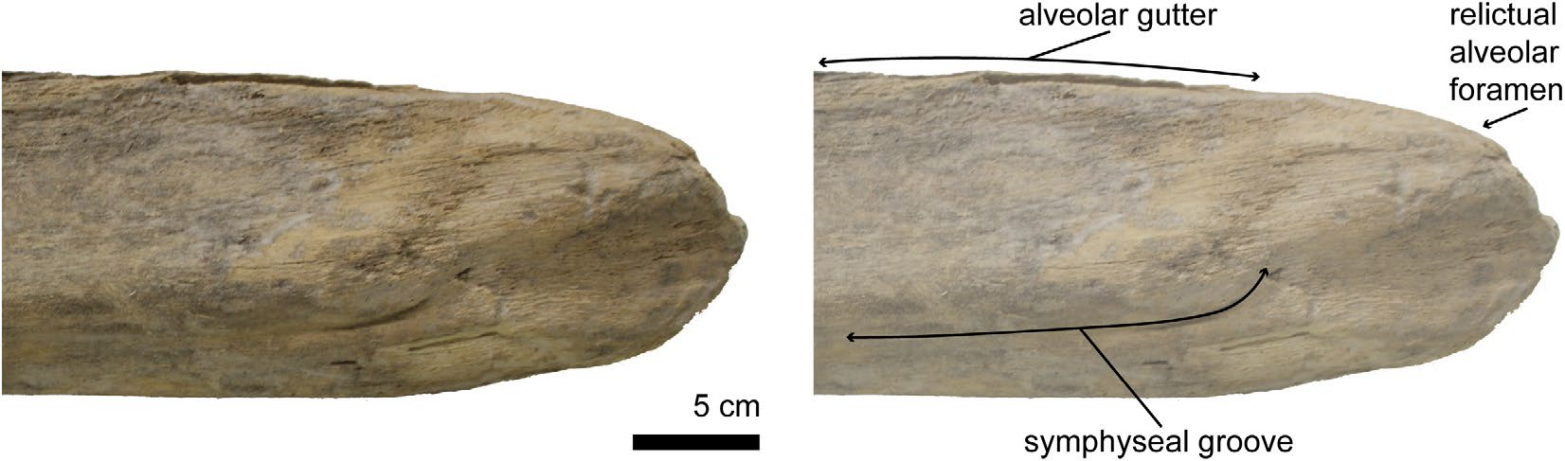
Symphyseal surface of the right mandible of *Balaenoptera acutorostrata* (UWBM 33258), reflected. The surface is completely smooth with a longitudinal groove (symphyseal groove).

All extant mysticetes have unfused, unsutured mandibles that do not touch anteriorly at the midline. This morphology likely permits considerable mobility at the joint, allowing mandibles to operate as individual levers in isolation with three axes of rotation (Lambertsen et al., 1995; Potvin et al., 2009; Werth et al., 2020). Here, we coin the term “decoupled” for this condition (i.e., unfused symphyses that are unsutured, leading to greater joint separation and independent multiaxial rotation). The symphyseal surface is completely smooth with a longitudinal groove (called the symphyseal groove) (Figure 7). Anterior to the symphyseal groove, the symphyseal surface is excavated and concave.

**FIGURE 8:**
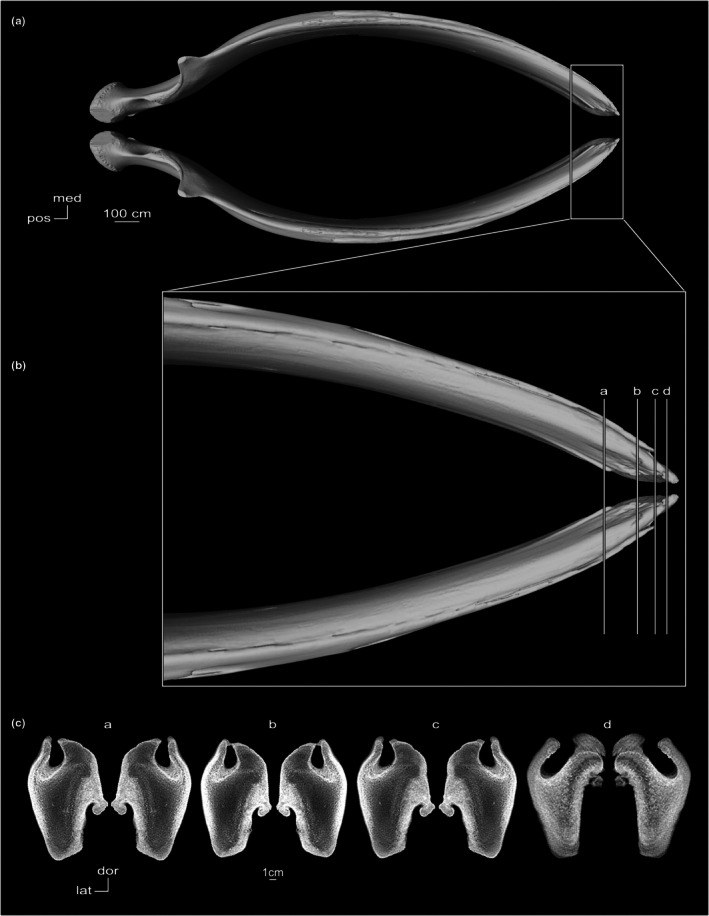
Left mandible of *Megaptera novaeangliae* (DFO 2408), reflected. (a) Dorsal view. (b) Close‐up of the symphysis in dorsal view showing the level of CT cross‐sectional slices (a–d). (c) CT coronal cross‐sectional slices (a–d).

CT coronal cross‐sections of *Megaptera novaeangliae* show a concave symphyseal surface in anterior view (Figure 8c). The alveolar gutter runs along the dorsal surface of the mandible and is most prominent in the anteriormost section (d), where it deepens and sweeps laterally (Figure 8c). The symphyseal groove divides the symphysis into two regions. The symphyseal surface of the dorsal region is dorsoventrally straight and overhangs the concave surface of the ventral region. Bone density of the mandible is low at the symphysis. In life, a mass of fibrous tissue fills the symphyseal space (Field et al., 2010).

The shape and orientation of the symphyseal groove differs across baleen whales. In *Balaenoptera* spp., the symphyseal groove is distinctly rectangular in shape, forming a shelf. In medial view, the groove begins a third of the way from the dorsal surface of the mandible in vertical orientation, passes ventrally, then turns almost 90‐degrees and bears posteriorly. In *Eschrichtius robustus*, the groove has a rounder shape and runs beneath a more bulbous overhang of the shelf. In *Balaena mysticetus* and *Eubalaena glacialis*, the groove originates at a level that is half way from the ventral surface of the mandible. Balaenids exhibit extreme torsion of the mandibles, in which the anteriormost portion of the mandible is laterally rotated (Bisconti, 2003) As a result, the symphyseal surface faces almost dorsally, and the labial surface of the mandible is oriented ventrally. This torsion is especially pronounced in *Eubalaena*, resulting in the dramatic overhang of the dorsally oriented symphyseal surface over a deeply excavated symphyseal groove.

Three of the observed fossil taxa exhibit decoupled mandibles with a smooth symphyseal surface and longitudinal groove (Table S6). In *Maiabalaena nesbittae*, the ventral portion of the symphyseal surface is excavated by a well‐developed symphyseal groove. In anterior view, the ventral margin of the mandible curls medially beneath the groove, forming a C‐shaped concavity ventral to the overhanging dorsal portion of the symphyseal surface. In *Mesocetus* sp. and *Parietobalaena palmeri*, the shape and orientation of the symphyseal groove are reminiscent of *Balaenoptera. Parietobalaena*, in particular, exhibits a groove that is strikingly rectangular in shape.

## 4 DISCUSSION

**FIGURE 9:**
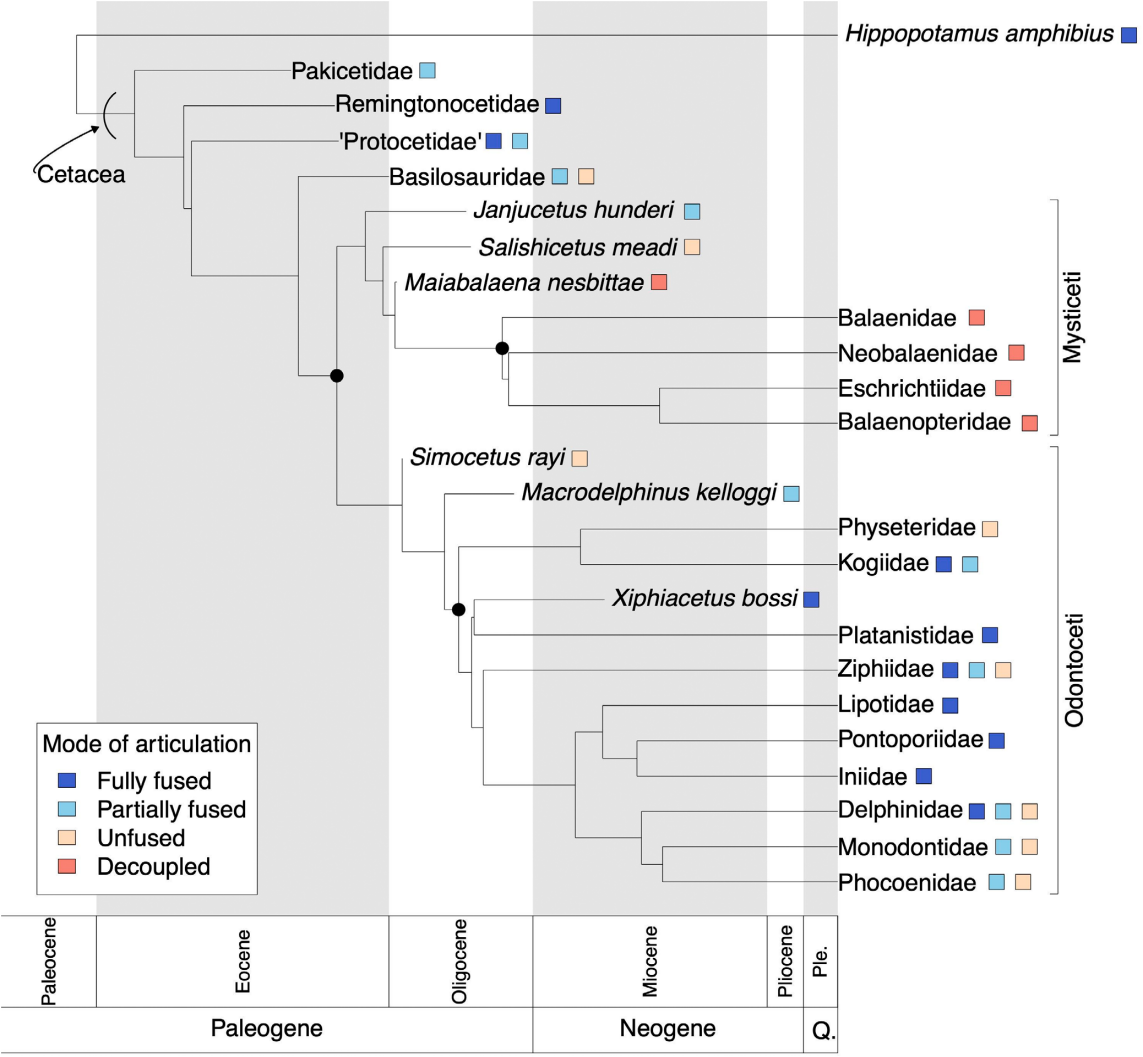
Distribution of different modes of articulation at the mandibular symphysis across whale phylogeny and geologic time. Colored squares indicate the mode(s) of articulation (fully fused, partially fused, unfused, or decoupled) observed for a particular taxon. Solid circles indicate crown‐based clades: Crown Cetacea, crown Mysticeti, and crown Odontoceti. Clade labels on the far right for Mysticeti and Odontoceti refer to the total group (i.e., include both stem and crown taxa). The phylogenetic relationships depicted here are based on a recent tree by Lloyd and Slater (2021). Note that *Hippopotamus amphibius* is included as a terrestrial artiodactyl outgroup. Cetacea includes all extinct lineages more closely related to crown cetaceans than *Hippopotamus*.

The diverse symphyseal morphologies observed in whales can be distributed across four broad modes of articulation (Table 2; Figure 9). As in terrestrial mammals, the morphology of the symphysis in odontocetes ranges from unfused to complete ossification. Mysticetes have evolved a decoupled symphysis, in which loose articulation of the mandibles confers greater mobility of the joint. Although an unfused symphysis is considered ancestral among mammals (Bhullar et al., 2019; Ravosa & Vinyard, 2020; Scapino, 1981; Scott et al., 2012), the fully decoupled symphysis in mysticetes is derived for both cetaceans and mammals at large.

**TABLE 2:** Modes of articulation at the mandibular symphysis in whales.

Mode	Unfused		Fused	
	Decoupled	Sutured		Fully fused
		Unfused	Partially fused	
Description	Smooth symphyseal surface. Mandibles do not touch. Longitudinal groove	Sutured. Smooth symphyseal surface	Sutured. Rugose symphyseal surface	Sutural obliteration (suture may no longer be visible). Mandibles cannot be disarticulated without breakage
Structure	*No synostosis*: Connected via fibrocartilage annulus with mucoid‐filled center in rorquals	*No synostosis*: Connected via fibrous tissue, ligaments, and a fibrocartilage pad wedged between bones	*Partial synostosis*: Connected via interlocking bony rugosities and calcified ligaments	*Complete synostosis (ankylosis)*: Firm, full fusion
Function	*Diarthrosis* (freely mobile)	*Amphiarthrosis* (slightly mobile)	*Synarthrosis* (immobile)	*Synarthrosis* (immobile)

### 4.1 Evolutionary context from the fossil record

Whales are descended from terrestrial artiodactyls (Gingerich, 2003; Graur & Higgins, 1994; Thewissen & Williams, 2002; Uhen, 2010). In many of their extant artiodactyl relatives, the symphysis is fused (Laws, 1968; Lee et al., 2019; Stover et al., 2017). *Pakicetus attocki*, one of the earliest whales from the Eocene of Indo‐Pakistan, exhibits a partially fused symphysis (West, 1980). During the Eocene, partial or full fusion is common among semiaquatic stem whales, such as remingtonocetids and protocetids (Kellogg, 1936; Thewissen & Bajpai, 2009). By contrast, pelagic stem whales, such as basilosaurids, exhibit less extensive fusion at the symphysis (Kellogg, 1936; Uhen, 2004). Our observations of both unfused and partially fused symphyses in *Zygorhiza kochii*, a basilosaurid from the latest Eocene, are consistent with this trend. In *Zygorhiza*, the anterior portion of the symphyseal surface is relatively smooth compared with the topography of the surface in partially fused symphyses (Figure 3a) and Scapino's Class II and III symphyses. The posterior portion of the symphyseal surface is more rugose compared with the anterior portion, with long striated ridges spanning the posterior two‐thirds of the surface. The posterior boundary is demarcated by a raised area with a rough texture, probably indicative of muscle attachment. In *Zygorhiza* specimens USNM PAL 16638 and USNM PAL 13774, a foramen or longitudinal sulcus perforating the symphyseal surface is preserved.

In the Oligocene, the emergence and establishment of crown lineages coincided with diversification in modes of aquatic feeding (Clementz et al., 2014; Marx & Fordyce, 2015; Pyenson, 2017; Slater et al., 2010). During this time, mysticetes evolved a decoupled, highly mobile symphysis that supported the evolution of aquatic suspension feeding (Deméré et al., 2008; Fitzgerald, 2012). Early diverging stem mysticetes with teeth exhibit partially fused or unfused symphyses (Barnes et al., 1994; Boessenecker et al., 2023; Fitzgerald, 2006, 2010, 2012; Lambert, Martínez‐Cáceres, et al., 2017). In *J. hunderi*, the symphysis is relatively short with a moderately rugose articulating surface (Fitzgerald, 2012). In *Salishicetus meadi*, the remarkably short articulating surface is completely smooth, and the posterior boundary is demarcated by a distinct longitudinal groove. In edentulous stem mysticetes from the Oligocene, the symphysis is decoupled (Boessenecker & Fordyce, 2016; Okazaki, 2012; Peredo et al., 2018; Peredo & Uhen, 2016). In *M. nesbittae*, the symphyseal surface is completely smooth with a deeply excavated symphyseal groove. Decoupled symphyses in stem mysticetes indicate that like tooth loss, the extreme condition observed in extant mysticetes evolved prior to the crown (Peredo et al., 2018). After the Oligocene, all crown mysticetes have a decoupled symphysis.

In *Simocetus rayi*, a stem odontocete from the early Oligocene, the symphyseal surface is conspicuously smooth and preserves a modest indentation that may represent a sulcus. Based on these observations, we follow previous authors in inferring an unfused symphysis (Fordyce, 2002). The overall texture and outline of the symphyseal surface, as well as the location of the longitudinal sulcus, are reminiscent of some extant delphinids, such as *Stenella*. The symphyseal surface is also reminiscent of some contemporary stem mysticetes from the Oligocene.

Full fusion coincides with an elongated symphysis in many of the Miocene odontocetes examined. However, these taxa vary in the overall shape and thickness of the symphyseal region. In some of the observed specimens, such as *Pomatodelphis*, the symphysis is dorsoventrally flattened. In *Isthminia*, dorsoventral and transverse thicknesses are more proportional. Unfused or partially fused symphyses are inferred in most of the fossil sperm whales examined. Mandibles of *Orycterocetus* sp. and an unnamed sperm whale from the Early Miocene (USNM PAL 489195) are unfused with modest topography of the symphyseal surface. In USNM PAL 489195, longitudinal ridges run along the symphyseal surface, as in extant giant sperm whales. The posterior boundary of the symphysis is indicated by a rough texture, whereas the distal portion of the symphyseal plate is comparatively smooth. Fully fused mandibles are observed in *Kogiopsis floridana*, a sperm whale from the Late Miocene of Florida.

### 4.2 Functional implications for feeding ecology

Here, we defined a decoupled symphysis as an unfused, unsutured symphysis that accommodates greater joint separation and independent multiaxial rotation. Decoupling of the symphyseal joint in baleen whales is a truly unique condition among mammals. The decoupled symphysis in baleen whales contributes to both functional and structural novelties associated with bulk filter feeding (Lambertsen et al., 1995; Pivorunas, 1977), including a sensory organ housed within the symphysis of rorquals (Pyenson et al., 2012). Anteaters (Vermilingua, Xenarthra) are perhaps the only other mammalian clade for which a loose, highly mobile symphysis has been documented (Ferreira‐Cardoso et al., 2020). Decoupled mandibles in baleen whales are also comparable to the kinetic jaws of macrostomatan snakes, in which mandibles can operate as independent levers in isolation (Gans, 1961; Kardong, 1977). Notably, in all three of these groups—baleen whales, anteaters, and snakes—an unfused symphysis is associated with enlargement of the oral cavity (Fitzgerald, 2012; Kardong, 1977; Naples, 1999).

The well‐developed posterior border of the symphysis in suction‐feeding odontocetes, such as *Physeter* and ziphiids, is consistent with the link between suction feeding and hyolingual musculature and function. Some suction feeders generate negative pressure by piston‐like retraction and depression of the tongue (Bloodworth & Marshall, 2007; Kane & Marshall, 2009; Werth, 2000, 2004, 2007). Tongue‐protracting muscles (i.e., genioglossus and geniohyoid) that originate on the posterior border of the symphyseal surface return the tongue to its original position (Werth, 2007; Werth & Crompton, 2023). Tongue protraction also contributes to expelling water in some suction‐feeding species (Werth, 2000). Stresses imposed by regular muscle engagement and use influence bone development at muscle attachment sites (Turcotte et al., 2022; White & Folkens, 2000). The prominence and arrangement of eminences on the posterior border suggest that some aspects of tongue function and mobility may be reflected in the morphology of the symphysis.

Our observations of extant and extinct longirostrine taxa (e.g., *Platanista* and *Xiphiacetus*) indicate that full fusion tends to coincide with extreme elongation of the symphysis. *Physeter* is a notable exception, exhibiting an elongated symphysis that is unfused. In river dolphins, such as *Platanista*, an elongated, fused symphysis may function as a long, fast lever for rapidly snapping up fast‐moving prey. This morphology is convergent with other aquatic tetrapods (Cuff & Rayfield, 2013; McCurry et al., 2017; Pierce et al., 2009; Walmsley et al., 2013). Because long levers are weaker and vulnerable to high strains (McCurry et al., 2017; McHenry et al., 2006; Walmsley et al., 2013), fusion may be necessary for strengthening and stabilizing the joint, especially in hyperlongirostrine odontocetes from the Miocene (McCurry & Pyenson, 2019).

Extant sperm whales are peculiar because the elongated, unfused symphysis may suggest a remarkably weak joint. However, sperm whales are suction feeders and do not rely on a functional dentition for preycapture (Werth, 2004). The structural weakness of the joint, as well as malformed jaws in mature specimens (Barroso et al., 2012), suggest that the symphysis may not play a significant role in feeding. Many fossil sperm whales, including inferred macroraptorial forms, exhibit an unfused or partially fused symphysis (Lambert, Bianucci, et al., 2017). Kogiids likely evolved fused symphyses after diverging from other physeteroids (Velez‐Juarbe et al., 2015). If an unfused symphysis represents the plesiomorphic condition for sperm whales, then unfused jaws in *Physeter* likely reflect a plesiomorphic state.

Modifications to the ancestral mammalian feeding apparatus supported the evolution of diverse modes of aquatic feeding in marine mammals (Marshall & Pyenson, 2019). In aquatic carnivorans, such as pinnipeds and sea otters (*Enhydra lutris*), the range of symphyseal morphology likely does not depart from the four classes of symphyses documented by Scapino (1981). However, feeding in an aquatic environment is mechanically different from feeding on land (Hocking et al., 2017; Marshall & Pyenson, 2019). As a result, functional and ecological explanations for the morphology of the symphysis in aquatic carnivorans may be different (Scott et al., 2012). In *Enhydra*, an unfused, flexible symphysis may facilitate shell‐cracking durophagy (Scapino, 1981; Tseng et al., 2016). In extant walruses, a fully fused symphysis may facilitate benthic suction feeding (Adam & Berta, 2002; Kastelein & Gerrits, 1990). Aquatic herbivores, such as sirenians and the extinct desmostylians, notably exhibit extensive fusion at the symphysis but differ in dorsoventral thickness and overall shape of the joint. In sirenians, the mandibular symphysis is fully fused, robust, and slightly downturned (Domning, 2018). In desmostylians, the overall symphyseal joint morphology is shovel‐shaped and the symphysis itself is dorsoventrally thin in cross‐section (RJS, pers. obs. *Desmostylus hesperus*; see Beatty, 2009; Domning et al., 1986; Uno & Kimura, 2004). Further investigation of the symphysis in these groups would provide insight into how phylogeny and ecology influence the form and function of the feeding apparatus in an aquatic environment.

### 4.3 Outstanding questions

Extensive symphyseal fusion may be adaptive or a response to the loads experienced by the joint during feeding (Scott et al., 2012). Fusion is likely adaptive in species that attain a fully fused symphysis prior to adopting an adult diet (Ravosa & Vinyard, 2020; Stover et al., 2017). Given the primacy of jaws in mammalian feeding, we suspect these assumptions can be applied across terrestrial, semiaquatic, and obligately aquatic mammals. However, the growth and development of the symphysis are unknown for the majority of cetacean taxa. Determining the timing of symphyseal fusion is therefore a necessary step in testing hypotheses about relative influences of selection and phenotypic plasticity on symphyseal morphology.

Several taxa exhibit intraspecific variation in the morphology of the jaw symphysis. For instance, unfused, partially fused, and fully fused mandibles were observed in *Ziphius*. Unfused mandibles were observed in calves and adult females, whereas partial or full fusion was observed in adult males. Like most sutures, the mandibular symphysis fuses with ontogenetic age, with considerable variation in the timing and degree of sutural closure across mammalian groups (Perrin, 1975; Scapino, 1981; Stover et al., 2017). As a result, some amount of ontogenetic variation is expected. In some beaked whales, such as *Mesoplodon*, mandibles are sexually dimorphic in size, shape, and dentition (Besharse, 1971; Macleod & Herman, 2004). More extensive fusion of the symphysis in adult males compared with adult females may be another instance of sexual dimorphism. Possible sexual dimorphism was also observed in *Physeter*, in which the symphyseal surface of adult males had a more complex topography compared with adult females. However, extant sperm whales exhibit extreme sexual dimorphism in body size (Nakamura et al., 2013; Whitehead, 2018). It is possible that apparent sexual dimorphism of the symphysis in *Physeter* is more directly linked to differences in body size (Scapino, 1981). Given the methodological and logistical limitations associated with large body size, habitat range, and conservation status (Pyenson, 2011), obtaining a large enough sample size poses a significant challenge for addressing questions about intraspecific variation. Nevertheless, future work can bear on these questions through further documentation of symphyseal morphology across different age and sex classes. These efforts can be augmented by implementation of CT and other internal imaging techniques that capture variation in the internal architecture of the symphysis, as well as opportunistic acquisition and study of fresh material from stranding occurrences.

## 5 CONCLUSION

Whales exhibit diverse morphologies of the mandibular symphysis, spanning the range of articulation observed in terrestrial mammals and evolving a novel, decoupled condition unique to mysticetes. The descriptions of the morphologies presented here are primarily based on gross anatomical observations of the osteology of the joint. Subsequent work should examine the histology of the symphysis and document the arrangement of soft tissue within the joint space. Further investigation of the vascularization and innervation of the symphysis across cetaceans could inform the origin of the sensory organ in rorquals. In addition to diverse modes of articulation uniting mandibles at the symphysis, we observed variation in the shape of the symphyseal surface. Future studies should investigate how the shape of the articulating surfaces relates to functional ecology. Explanations for intraspecific variation, such as ontogeny and sexual dimorphism, also warrant further investigation. Finally, future work should examine the range of morphological and functional diversity in the mandibular symphysis of other marine mammals.

## AUTHOR CONTRIBUTIONS

All authors evaluated and analyzed the data, reviewed and edited drafts of the article, and contributed to preparation of figures and/or tables. RJS and CMP conceived and planned the project. RJS examined all of the specimens in the study, conducted the comparative and descriptive anatomical work, and wrote the original draft. CMP modeled digital data. NDP provided access to specimens at the Smithsonian Institution's National Museum of Natural History.

## FUNDING INFORMATION

Study of Burke Museum specimens was supported by the Burke Museum Vertebrate Paleontology Collection Study Grant. Study of specimens in collections at the Smithsonian Institution's National Museum of Natural History was supported by the Remington Kellogg Fund.

## Supporting information


Data S1.

## Data Availability

The data that supports the findings of this study are available in the supplementary material of this article.
